# Philadelphia Alms-House Hospital, Blockley

**Published:** 1842-01-15

**Authors:** 


### CLINICAL REPORTS.

### Philadelphia Alms-House Hospital, Blockley.

*Congenital want of power, and muscular atrophy of the lower extremities.*—P. T., black, æt. 50, entered the hospital Nov. 24th. Of his history I know nothing, except that he was accustomed to move about the streets in a little waggon, propelled by himself, having been unable to walk from childhood. His head was large, his neck, chest, and superior extremities remarkably well developed; his inferior extremities flexed, and very much atrophied.

I did not see the patient until the day of his death; and no notes of his case were taken. Consequently, I can give but an imperfect account of his medical history. Previously to his last attack he had enjoyed good health; after recovering from a cough which had lasted some months, and which, as we shall see, depended upon a tuberculous deposit.

At the time of his attack he complained of inability to retain food on his stomach, and had been thus affected for three days. He had no jaundice; he was cheerful and his mind was clear. He took that night opii gr. ss. hyd. chlor. mit. gr. x. This operated twice on his bowels, and in the morning he appeared better. On the evening of the 27th, the nausea returned, and was relieved by a draught containing twenty drops of the tincture of opium. On the morning of the 28th, the sickness of stomach returned, and an emetic of ipecacuanha was administered. In the evening his intellect was disturbed, and nervous tremors were present. The conjunctiva presented the tint of slight jaundice, pulse small and feeble, about 100 in the minute. On being questioned on this point, he said that he had been drinking about a pint of spirit a day for some time before his entrance, (an ounce of brandy every two hours.)

On the morning of the 29th, he offered the following symptoms: insensibility to sound, and almost complete coma; incessant hiccough, alternating with moaning; conjunctiva deeply jaundiced and injected; pupils much contracted; grimaces, especially a drawing of the mouth to the left; tremors and subsultus of the left arm, with strong contraction and rigidity; left leg participating in the tremors; sensibility to strong impression on the skin; some contraction of the right arm, but no contractile sensibility remaining; rattle commencing in the trachea; pulse very small, scarcely perceptible, about 60.

℞. Spts. Vin. Gallic. ℥ss. q. ¼ h.

Ammon. carb. gr. v. q. ½ h.

Enema Ol. Terebinth, q. h. 4 ta.

Emp. vesicat. ad caput.

Sinapism ad abdomen and femora.

No reaction took place; he gradually sank, and died at six P. M.

*Autopsy, eighteen hours after death, results as follows:*

*Head.*—Scalp of a deep yellow colour, its vessels containing much blood; vessels between the dura mater and cranium tinged with blood more red than usual; dura mater of a yellowish pink colour, presenting a punctiform injection at the junction of the sagittal and coronal sutures, and along the inferior margin of the parietal bones; arterial vessels full, and the minute branches conspicuous; effusion of serum into the cavity of the arachnoid to the amount of three fluid ounces; arachnoid somewhat opaque in parts, and distended with serum effused under it. Pia mater presented a minute capilliform injection through its whole extent, more marked at the base of the brain. Central substance, both cortical and medullary, natural in colour and consistence; ventricles contained but a few drops of yellowish fluid, lining membrane healthy.

*Thorax.—Lungs*—Strong adhesions of a remote date at the summit of the right lung, slighter ones at corresponding portion of the left; traces of emphysema in both. At the summit of the right lung, a cavity of the size of a walnut, with firm walls and a smooth lining membrane; this was empty and communicated with a large bronchus. The left lung near its summit, and posteriorly, presented a slight depression, at which point its tissue was firm and cartilaginous to the feeling, and seemingly free from tubercular matter; this appeared to be the cicatrix of a cavity. Both lungs contained a moderate number of small tubercles, some semicretaceous, others very hard and imbedded in a fibrous tissue. Posteriorly the lower lobes were much congested and softened, and in a state resembling apoplexy.

*Heart.*—Slight effusion into the pericardium ; heart itself rather larger than the average ; parietes normal in thickness but softened ; slight traces of previous endocarditis in the left ventricle. In the right ventricle was found a semi-organized coagulum of a yellow colour, extending in one direction into the auricle, and an inch or two into the descending cava; in the other direction into the pulmonary artery and its branches to the most minute ramifications traceable by the naked eye. When drawn out and suspended in water, its terminating filaments resembled the radicles of a plant. A coagulum less organized was found in the left ventricle, extending through the arch of the aorta and a short distance in the subclavian and carotid arteries.

*Abdomen.*—Beneath the skin was a layer of fat an inch in thickness. The omentum contained also a great quantity of it.

*Stomach* contained a thick tenacious dark coloured mucus. Its interior surface was of a dusky pink colour, and, near the cardiac orifice was a bright arborescent injection. Scattered over the surface were slight depressions, the commencement of follicular ulceration. The mucous membrane was thickened and softened, being easily removed in strips of six or eight lines in length.

*Intestines.*—The duodenum offered traces of active inflammation, extending from the pyloric orifice below the orifice of the ductus choledochus communis. The ileum and jejunum were healthy, and contained a dark brown thick mucus. Peyer's glands conspicuous, but not elevated. The colon contained some thin, dark, fecal matter. On its mucous membrane were seen small cicatrices, and a few ulcers in the process of cicatrization.

*Liver* of a yellowish red or brickdust colour, slightly cyrrhosed; the surface of the lobulus spigelii and the adjoining portion of the right lobe presented a stellated injection of a dark colour. The parenchyma was considerably softened.

The *gall-bladder* contained some thick, dark bile; as did also the hepatic, and cystic, and common ducts; they offered no trace of inflammation.

*Spleen* of a middle size, red and softened.

*Pancreas* firm and of a light colour.

*Kidneys.*—These were enveloped in a thick covering of fat; they were pale and softened.

*Spinal Marrow.*—Only the lower portion was examined ; its substance normal in consistence and colour. The pia mater offered a bright and minute venous injection.

*Nerves of the lower extremities.*—The sciatic and anterior crural nerves were of the full size, and nowise altered from the normal condition; but the external iliac and femoral arteries were very small, the latter being scarcely larger than the brachial. The thigh was but ten inches in circumference, and consisted in a great measure of fat, forming a layer an inch and a quarter thick under the skin. Muscles of the thigh were thin and pale; the sartorius being with difficulty distinguished from the surrounding fat—and the rectus internus not more than a line in thickness. The leg was but eight inches in circumference at the calf. H. S.

